# Selection of yeasts and lactic acid bacteria to improve the nutritional quality, volatile profile, and biofunctional properties of chickpea flour

**DOI:** 10.1016/j.crfs.2025.101204

**Published:** 2025-09-23

**Authors:** Solidea Amadei, Irene Gandolfi, Davide Gottardi, Chiara Cevoli, Margherita D'Alessandro, Lorenzo Siroli, Rosalba Lanciotti, Francesca Patrignani

**Affiliations:** a Department of Agricultural and Food Sciences, Campus of Food Science, Alma Mater Studiorum, University of Bologna, Piazza Goidanich 60, 47521, Cesena, Italy; b Interdepartmental Centre for Agri-Food Industrial Research, Campus of Food Science, Alma Mater Studiorum, University of Bologna, Via Quinto Bucci 336, 47521, Cesena, Italy

**Keywords:** Chickpea flour, Yeasts, Fermentation, Protein content, Sugar content, Volatile molecule profile, Antioxidant activity, Prebiotic activity

## Abstract

The growing global demand for dietary protein calls for alternative sources. Chickpeas (*Cicer arietinum* L.) are a source of proteins and bioactive compounds, but their digestibility is limited by antinutritional factors and raffinose-family oligosaccharides (RFOs). Fermentation with lactic acid bacteria (LAB) can improve nutritional quality by enhancing protein bioavailability, reducing antinutritional compounds, and boosting antioxidant activity. However, at the top of our knowledge, no information is present regarding the impact of yeasts. This study assessed how different yeasts and LAB affect chickpea flour properties. Microbial growth, soluble protein and peptide content, sugar composition, phytate levels, antioxidant and prebiotic activity, and volatile molecule profiles were analyzed. Addition of the tested microorganisms reduced high-molecular-weight proteins into peptides and reduced RFOs in a strain-dependent fashion, particularly with *Lacticaseibacillus paracasei* L and *Saccharomyces cerevisiae* FB2. Instead, samples with *Debaryomyces hansenii* Y15A and Y17A promoted probiotic growth during early incubation stages. Volatilome analysis revealed modified aroma profiles, with aldehydes and esters reaching values below the limit of detection in specific samples. LAB-fermented samples showed the highest antioxidant activity, while yeast-fermented samples, particularly with *D. hansenii* Y15A, significantly lowered phytic acid levels (up to 0.82 g/100 g). These results demonstrate that yeasts can also enhance the nutritional and biofunctional properties of chickpea flour, making it a promising ingredient for high-protein functional foods.

## Highlights

• Fermentation enhances chickpea flour's nutritional and biofunctional properties.
• Yeast and LAB strains reduce antinutritional factors in a strain-specific fashion.
• Fermented flour showed prebiotic activity supporting probiotic growth.
• SPME-GC-MS analysis revealed a reduction in beany-flavor volatile compounds and enrichment of aroma-active molecules.

## Graphical abstract

**Figure:**
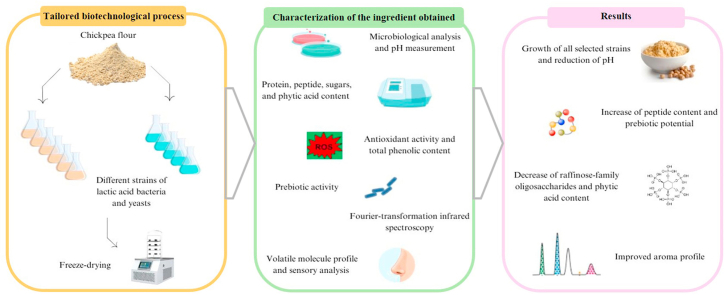

## 1 Introduction

In recent decades, the global demand for dietary protein has been rising steadily, driven by population growth and increasing prosperity. According to the Food and Agriculture Organization of the United Nations (FAO), the production of animal-based protein sources is unlikely to grow significantly in the coming years. While the demand for animal-based proteins remains consistently high, they are often considered less environmentally sustainable. Therefore, to promote more sustainable eating habits, reducing reliance on animal-derived proteins is encouraged. Alternative protein sources must be explored to meet the nutritional needs of the population (FAO, 2021). In this regard, plant-based proteins may offer greater health benefits than their animal counterparts. For example, consuming plant proteins has been associated with lower all-cause mortality and a reduced risk of cardiovascular diseases (Langyan et al., 2022).

Legumes, such as beans, chickpeas, peas, lentils, and lupines, are an excellent source of dietary protein, providing between 17 % and 46 % protein content. Regular consumption of these products has been associated with a reduced risk of cardiovascular diseases, obesity, high serum cholesterol, and diabetes (Arbab Sakandar et al., 2023). For this reason, legume flours are increasingly being incorporated into food formulations to enhance the nutritional value of products, particularly by boosting their fiber and protein content.

Among legumes, chickpea (*Cicer arietinum* L.) is the second most-produced legume globally, with an annual production of 9.96 million tons, accounting for 13 % of total legume production. It is widely grown and consumed around the world (FAO, 2021). Chickpeas are composed of approximately 61–62 % carbohydrates, 17–21 % protein, 9–12 % water, 5–7 % fat, and 3 % ash. Their main protein fractions include globulins (53–60 % of the total protein content), glutenins (19–25 %), albumins (8–12 %), and prolamins (3–7 %) (Day, 2013). Additionally, chickpeas contain bioactive compounds such as phenolic acids and isoflavones (Rachwa-Rosiak et al., 2015). Compared to wheat flour, chickpea flour is richer in protein, fiber, and polyunsaturated fatty acids. Its use in food formulations is gaining popularity worldwide due to its beneficial technological properties, wide availability, and suitability for vegan and gluten-free diets. Studies have already reported improvements in protein content, nutritional value, and sensory properties when chickpea flour is incorporated into food products (Dandachy et al., 2019), (Garcia-Valle et al., 2021). However, like other legumes, chickpeas are deficient in sulfur-containing amino acids, such as methionine and cystine, and contain antinutritional factors, including trypsin and chymotrypsin inhibitors, phytic acid, and tannins, which can limit their consumption (Grasso et al., 2022). Furthermore, chickpeas are rich in raffinose-family oligosaccharides (RFOs), which are known to cause flatulence in humans and animals (Elango et al., 2022), with stachyose constituting approximately 61 % of these oligosaccharides (Rachwa-Rosiak et al., 2015).

Biotechnological approaches using selected safe microorganisms are increasingly applied to plant-based foods to improve their organoleptic, nutritional, technological, and functional properties (Sáez et al., 2022).

Fermentation extends shelf life, enhances protein bioavailability, and enriches aroma compounds while reducing antinutritional factors (Angulo-Bejarano et al., 2008). It also increases the total phenolic content of the flour matrix, boosting its antioxidant activity (Sáez et al., 2022). Furthermore, fermentation enhances the quality and functionality of chickpea proteins by breaking them down into smaller polypeptides, free amino acids, and bioactive peptides, thereby improving their essential amino acid profile and nutritional value (Emkani et al., 2022). Additionally, fermented chickpea exhibits superior oil absorption capacity, foaming properties, and foam stability compared to untreated flour or flour subjected to alternative treatments such as soaking, germination, or cooking (Chandra-Hioe et al., 2016). It is important to note that these modifications can be highly dependent on the genus, species, and strain of the microorganisms used, highlighting the need for careful strain selection in biotechnological applications. Most recent studies on chickpea fermentation have focused specifically on lactic acid bacteria (LAB), particularly due to their safety status and effectiveness in improving nutritional and functional attributes. Examples include the works of (Sáez et al., 2022), (Chiacchio et al., 2025), (Zhou et al., 2025), (Hurtado-Murillo et al., 2025), (Parmigiani et al., 2025), all of which report significant enhancements in protein digestibility, antioxidant activity, and sensory properties following lactic fermentation. However, the literature regarding the use of yeasts is scarce. Yeasts are important microorganisms involved in various fermentation and ripening processes of both traditional and novel foods, where they can induce significant modifications in the final product (Tofalo et al., 2020). For all these reasons, the aim of this study was to evaluate the impact of selected strains of yeasts, in comparison with different species and strains of LAB, on the fermentation of hydrated chickpea flour to improve nutritional quality, volatile profile, and biofunctional properties of the final ingredient. To attain this goal, in addition to monitoring the microbial growth during fermentation, freeze-dried samples were analyzed for their soluble protein concentration and profile, peptide content, sugar composition (glucose, sucrose, and RFOs), phytate levels, antioxidant and prebiotic activity, and total phenolic content. Moreover, the products were analyzed for their volatile molecule profile and odour acceptability.

## 2 Materials and methods

### 2.1 Growth conditions of yeast and bacteria strains

All the 11 microbial strains applied in this work belong to the culture collection of the Department of Food Science, Alma Mater Studiorum – University of Bologna (Italy) and were isolated from different environments such as: Po River lagoons, dairy products, refrigerated foods, wine lees and dry-aged meat. Specifically, 6 lactic acid bacteria (LAB) strains and 5 yeast strains were selected and listed in Table 1. Before their use, yeasts were cultured twice in Yeast extract Peptone Dextrose (YPD) broth (Oxoid, Basigstone, UK) and, among the strains, *Y. lipolytica* and *D. hansenii* strains were incubated in agitation, at room temperature for 64 h while, *S. cerevisiae* strain was incubated at 30 °C for 24 h. LAB were cultured twice in MRS broth (Oxoid, Basigstone, UK) for 24 h at 37 °C. Cultures were centrifuged at 5000 rpm for 10 min and the biomasses were resuspended in the same volume of saline solution (NaCl, 9 g/L) before their addition in the hydrated chickpea flour.

**Table 1:** Microbial strains used in the study, environment of origin and growth conditions applied.

Microorganism	Strain	Origin	Growth conditions
*Lactococcus lactis*	LBG2	Cow milk	M17, 30 °C, static anaerobic condition
*Latilactobacillus sakei*	M12A	Dry-aged meat	MRS, 30 °C, static anaerobic condition
*Latilactobacillus curvatus*	BS3	Kefir	MRS, 30 °C, static anaerobic condition
*Lactiplantibacillus plantarum*	LP82	Caciotta cheese	MRS, 37 °C, static anaerobic condition
*Lactiplantibacillus plantarum*	LP23	Wine lees	MRS, 37 °C, static anaerobic condition
*Lacticaseibacillus paracasei*	L	Wine lees	MRS, 37 °C, static anaerobic condition
*Debaryomyces hansenii*	Y17A	Dry-aged meat	YPD, 25 °C, agitation aerobic condition
*Debaryomyces hansenii*	Y15A	Dry-aged meat	YPD, 25 °C, agitation aerobic condition
*Yarrowia lipolytica*	PO17	Po river	YPD, 25 °C, agitation aerobic condition
*Yarrowia lipolytica*	Y3	Refrigerated foods	YPD, 25 °C, agitation aerobic condition
*Saccharomyces cerevisiae*	FB2	Sourdough	YPD, 30 °C, static aerobic condition

### 2.2 Preparation of hydrated chickpea flour

Commercial chickpea flour (Molino Maraldi, Cesena, Italy) was applied in this work. The proximal composition of the flour per 100 g and reported on product label was: carbohydrates 47 g (of which sugars 4 g), fats 6.3 g (of which saturated fatty acids 0.9 g), proteins 21 g, fibers 13.6 g, salt 0.015 g. The flour was used as such without any pretreatment. For each microorganism, 100 g of chickpea flour were mixed with 200 ml of bottled water (1:2 w:w). The mix was divided into three sterile flasks (*n* = 3) and inoculated with the respective microorganism at an initial concentration of approximately 5 and 6 log CFU/g for yeasts and LAB, respectively. The incubation was carried out for 72 h in shaking conditions (150 rpm) at room temperature for samples inoculated with yeasts and for 48 h at 30 °C for samples inoculated with LAB and *S. cerevisiae*. A control sample, prepared without any added inoculum, was collected immediately after formulation and used as 0 h. During fermentation, samples were taken at 24 and 48 h, or 24, 48, and 72 h for LAB and yeasts quantification, respectively, and pH measurements. At the end of fermentation (48 or 72 h), samples were immediately processed for microbiological analyses, pathogen detection and odour evaluation. The remaining sample was stored at −80 °C. Six g of samples were then used for volatilome analysis, while the rest underwent lyophilization, which was carried out for five days using a Drywinner Heto freeze-dryer (Cambridge Biosystems, Cambridge, UK).

### 2.3 Microbiological analyses and pH measurements

Microbiological analyses of the samples inoculated with LAB and *S. cerevisiae* were performed after 0, 24, and 48 h, whereas for samples inoculated with the other yeasts, sampling was performed after 0, 24, 48, and 72 h of fermentation by plate counting, using 1 mL of the sample subjected to serial decimal dilution with saline water (NaCl, 9 g/L). Then, all the dilutions were plated on specific media: YPD (Oxoid Ltd) agar with 0.02 % chloramphenicol for the enumeration of yeasts; MRS (Oxoid Ltd) agar with 0.02 % cycloheximide for LAB, *Bacillus cereus* selective agar base (MYP) for *Bacillus cereus*, and Violet Red Bile Glucose Agar (VRBGA, Oxoid Ltd) for *Enterobacteriaceae*. Plates were incubated for 24–48 h at 30 °C for MYP and YPD, respectively, 24–48 h at 37 °C for VRBGA and MRS, respectively. The presence/absence of *Listeria monocytogenes* and *Salmonella* spp. was performed on the samples at the end of fermentation according to ISO 11290–1:2017 and ISO 6579–1:2017, respectively. Pathogens were not present in all the samples. All the cell counts are the mean of three independent biological replicates (*n* = 3). pH measurements were performed on all samples during fermentation with 24-h intervals using a pH-meter (Seven Compact, Mettler Toledo, USA).

### 2.4 Protein characterization

#### 2.4.1 Protein extraction

Protein extraction was carried out on the freeze-dried samples according to (Siroli et al., 2022) with some modifications. More specifically, 50 mM Tris-base buffer solution at pH 8.5 was used to solubilize the proteins. The sample diluted with buffer (ratio 1:10 w/v) was mixed for 60 min at room temperature and then centrifuged at 15000 rpm for 10 min. The supernatant was filtered with Wathman filter (0.4 μm) and stored at −20 °C until analyses. These extracts were used for protein and peptide quantification.

#### 2.4.2 Protein content

Soluble protein quantification in each sample was performed as (Gottardi et al., 2022), using the Bradford protein assay kit (Bio-Rad; Hercules, CA, USA) following the manufacturer's instructions. As a reference, a calibration curve was obtained using different concentrations of bovine serum albumin (BSA, Bio-Rad Laboratories, Milan, Italy) as standard (0.025–1.0 mg/mL). The BSA calibration curve equation was used to calculate the protein content in fermented chickpea samples. The trial was performed on the three biological replicates (*n* = 3).

#### 2.4.3 Proteolysis profile

Protein profile of the samples was obtained by sodium dodecyl sulphate-polyacrylamide gel electrophoresis (SDS-PAGE) according to (Gottardi et al., 2023) with some modifications. 10 mg of freeze-dried chickpea flour sample was dissolved in 125 μL of a solution consisting of 50 % of sterile distilled water and 50 % of Laemmli Sample Buffer 2X (Bio-Rad Laboratories, Milan, Italy) containing β-mercaptoethanol. The mixtures were incubated at 100 °C for 5 min and centrifuged at 13600 rpm for 10 min. Supernatants (10 μL) were charged on a 8–16 % Criterion TGX precast Gel (Bio-Rad Laboratories, Milan, Italy). 5 μL of Precision Plus Protein Standard All Blue (Bio Rad) were used as standard. Gel was run in a Mini Protean Cell System with a Tris-Glycine SDS Running Buffer at 50 V for the first 10 min and at 100 V for 1 h. Gels were stained with Coomassie Blue for 2 h in agitation and de-stained for 2 h with the de-staining solution (40 % glacial acetic acid, 10 % methanol). Pictures were taken with Bio-Rad's GS-900 (Bio-Rad, Milan, Italy).

### 2.5 Peptide content

Peptide content in each sample was determined by the o-phthalaldehyde (OPA) method according to (Church et al., 1983) with some modifications. The extracted sample (50 μL), previously diluted with distilled water (1:50), was added to the OPA solution (950 μL) and incubated for 2 min in the dark. The absorbance was measured at 340 nm with the UV–visible spectrophotometer (UV-1800, Shimadzu, Kyoto, Japan). As a reference, a calibration curve was obtained using different concentrations of serine as standard (0.1–1.5 mg/mL). The serine calibration curve equation was used to calculate the peptides content in fermented chickpea samples. The trial was performed on three biological replicates (*n* = 3).

### 2.6 Sugars content

To determine the concentration of sugars in the fermented chickpea samples, the Megazyme (Megazyme International Ireland Limited, Bray, Ireland) kit K-RAFGL 08/23 raffinose/sucrose/glucose assay was applied. Briefly, 0.5 g of sample was first resuspended in 5 mL of ethanol (95 % v/v) and then incubations and buffer additions followed the manufacturer's instructions. Glucose, sucrose and RFOs concentrations were determined by measuring the absorbance at 510 nm with the UV–visible spectrophotometer (UV-1800, Shimadzu, Kyoto, Japan) and expressed in mmol/100 g. Water and glucose (included in the kit) were used as negative and positive controls, respectively. Sugar content was calculated based on the assay protocol provided by the manufacturer. The trial was performed on three biological replicates (*n* = 3).

### 2.7 Phytate content

To detect the phytase activity, an *in vitro* assay was performed, incubating fresh cultures with phytic acid, according to (Singh et al., 2013), with some modifications. Briefly, the tested strains were centrifuged, and the pellet resuspended directly in the test solution (1.5 % glucose, 0.5 % (NH_4_)_2_SO_4_, 0.01 % NaCl, 0.05 % KCl, 0.001 % FeSO_4_, 0.01 % MgSO_4_.7H_2_O, 0.01 % CaCl_2_.2H_2_O, 0.001 % MnSO_4_, pH 6.5 with 0.5 % calcium phytate). At the end of incubation (96 h at 30 °C) the supernatant was used to measure the phytate content. For the food matrices, samples were extracted with hydrochloric acid (0.66 M) according to the Megazyme kit K-PHYT 05/19 Phytic Acid (Phytate)/Total Phosphorus Assay (Megazyme International Ireland Limited, Bray, Ireland), following the manufacturer's instructions. After the enzymatic dephosphorylation reaction, the determination of phytate content was performed using a UV–visible spectrophotometer (UV-1800, Shimadzu, Kyoto, Japan). A calibration curve was generated using the phosphorus standards provided in the kit (ranging from 0.5 to 7.5 μg), and phytate concentration was calculated using the formula supplied by the manufacturer. Water was used as blank. Analyses were conducted on three biological replicates (*n* = 3).

### 2.8 Antioxidant activity and total phenolic content

The antioxidant activity (DPPH and ABTS) and total phenolic content were evaluated on the extracted samples according to the methodology reported by (Siroli et al., 2022), (Slinkard and Singleton, 1977). For both assays, standard curves were generated using Trolox at concentrations ranging from 1.95 to 1000 mg/L for DPPH, and from 5 to 400 mg/L for ABTS. Water was used as blank. The absorbance of the DPPH and ABTS working solutions was adjusted to 1.10 ± 0.20 Abs and 0.70 ± 0.02 Abs, respectively. The trial was performed on three biological replicates (*n* = 3).

### 2.9 Prebiotic activity

The prebiotic activity of the samples was determined according to the methodology reported by (Braschi et al., 2024) with minor modifications. The probiotic strains tested were *Bifidobacterium longum* subsp. *infantis* DSM 20088 and *Lacticaseibacillus rhamnosus* GG, that were cultured in MRS with 0.05 % cysteine for 24 h at 37 °C. Survival in SIF was monitored in response to not-inoculated and inoculated samples after 3, 6, and 24 h by plate counting on MRS with 0.05 % cysteine that were incubated at 37 °C for 24 h, anaerobically or aerobically, respectively for *B. longum* subsp. *infantis* DSM 20088 and *L. rhamnosus* GG. The trial was performed on three biological replicates (*n* = 3).

### 2.10 Volatile molecule profile

The volatile molecule profiles were detected at the end of the incubation period (i.e. after 48 h for samples inoculated with *L. paracasei* L, *L. plantarum* LP23 and LP82, *L. lactis* LBG2, *L. sakei* M12A, *L. curvatus* BS3 and *S. cerevisiae* FB2, and after 72 h for samples inoculated with *Y. lipolytica* Y3 and PO17 and *D. hansenii* Y15A and Y17A). To evaluate modifications due to the fermentation process, a not inoculated sample (control) was analyzed. Two analytical replicates were analyzed for each condition (*n* = 2) with SPME/GC-MS technique. All the samples contained an internal standard (IS: 4-methyl-2-pentanol) at a final concentration of 2 mg/L that was used to calculate the concentration of the single molecules expressed in mg/L equivalent to the IS according to (Rossi et al., 2024).

### 2.11 Fourier-transformation infrared spectroscopy (FTIR)

FT-IR spectra were acquired by using a Tensor 27 TM FTIR spectrometer system (Bruker Optics, Ettlingen, Germany) at room temperature. The system was equipped with a Rocksolid interferometer and a DigiTect detector system, coupled to an attenuated total reflectance accessory (ATR, PIKE Technologies, Madison, WI, USA). A small amount of sample was placed on the ATR surface, and by using a pressure clamp, a thin layer was obtained. Spectra were acquired (32 scans sample) in the range of 4000-700 cm^−1^ at a Fourier transform (FT) resolution of 4 cm^−1^. The absorbance spectrum was collected against a background obtained with a dry and empty ATR cell. Three spectra were recorded for each sample, the ATR crystal was cleaned with a cellulose tissue soaked in n-hexane and then rinsed with acetone.

### 2.12 Preliminary odour evaluation

At the end of the incubation period, inoculated samples were evaluated for their odour characteristics and compared to the control sample, following a procedure similar to that described by (Chen et al., 2018), (Guardado-Félix et al., 2020). A odour evaluation was conducted by a group of 20 participants, recruited from staff members and research fellows at the Department of Agricultural and Food Sciences, Alma Mater Studiorum University of Bologna. Participants were fully informed about the study's purpose, procedures, and any potential risks, and gave their verbal consent to participate. No personal data were collected, and the evaluation was conducted anonymously as part of an informal internal survey. The procedure involved only olfactory assessment, with no tasting or ingestion of the samples. Evaluation steps included: development of a sensory vocabulary, construction of a scoring sheet, distribution of coded, odour-only samples in closed cups, explanation of the experimental objectives to the participants, and individual scoring of the perceived intensity of selected odour attributes. Data were analyzed using spider plots. A 10-point line scale was used, ranging from “extremely disliked” (1) to “extremely liked” (10). Olfactory intensity, herbaceous hint, legume hint, fermented odour, acidity, persistence, perception of anomalous odours, and olfactory pleasantness were considered for the olfactory evaluation.

### 2.13 Statistical analysis

The significance differences between the means of all samples were evaluated using one-way ANOVA followed by Tukey HSD Post-Hoc Test at *p* < 0.05 (STATISTICA v. 8.0; StatSoft, Tulsa, OK, USA). Pearson correlation analyses were also performed between volatile compound data and odour parameters, as well as between antioxidant activity and total phenolic content, in order to assess potential associations among these variables (Microsoft Excel, 2021). Staring from volatile compounds (autoscaled values), principal component analysis (PCA) was adopted to evaluate the influence of the volatile molecules on sample behaviour. Concerning the FT-IR spectra, the region between 2750 and 1850 cm^−1^ was deleted as it contained no useful information, but only instrumental noise. Subsequently, spectra were smoothed (Savitzky-Golay method, 7 points) to reduce noise. The absorbance data were normalized by using the Standard Normal Variate (SNV) and pre-treated by first derivative (Savitzky-Golay method, 7 points) and finally mean cantered (MC). PCA analysis was used as an explorative technique to visualize the samples according to treatment type. To minimize the risk of overfitting, all PCA were subjected to cross-validation using the Venetian blinds approach. Data Elaboration was performed with PLS Toolbox for Matlab2018a®.

## 3 Results and discussion

### 3.1 Microbiological characterization and pH

**Fig. 1:**
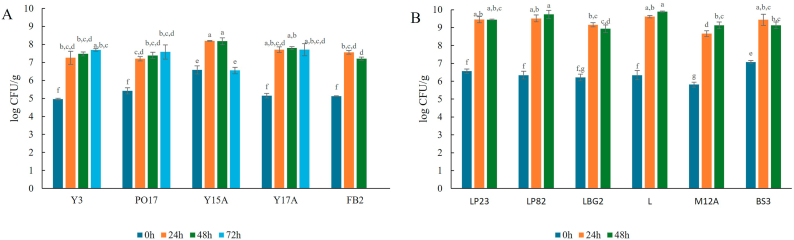
Cell loads (log CFU/g) of yeasts (A) or LAB (B) inoculated in chickpea flour. The strains tested were *Y. lipolytica* Y3 (Y3), *Y. lipolytica* PO17 (PO17), *D. hansenii* Y15A (Y15A), *D. hansenii* Y17A (Y17A) and *S. cerevisiae* FB2 (FB2), for yeasts and *L. plantarum* LP23 (LP23), *L. plantarum* LP82 (LP82), *L. lactis* LBG2 (LBG2), *L. paracasei* L (L), *L. sakei* M12A (M12A) and *L. curvatus* BS3 (BS3) for LAB. Cell loads were determined before incubation (0 h) and after 24, 48 h incubation for LAB or 24, 48 and 72 h for yeasts. The results are the mean of three independent replicates (*n* = 3) ± standard deviation.

Microbiological analyses were conducted to assess the growth of the inoculated microorganisms throughout the incubation period, which lasted 48 h for LAB and *S. cerevisiae*, and 72 h for the other yeast strains. As shown in Fig. 1A, all yeast strains (starting from around 5 log CFU/g) exhibited growth in the matrix. *Y. lipolytica* Y3, PO17, *D. hansenii* Y17A and *S. cerevisiae* FB2, reached maximum 7 log already after 24 h and they maintained this concentration throughout the entire incubation period. On the other hand, *D. hansenii* Y15A, which was inoculated at higher concentration (6 log CFU/g) reached 8 log CFU/g after 24 h. However, a reduction of the strain of around 2 log was observed after 72 h incubation. Also, all the tested lactic acid bacteria grew on the substrate (Fig. 1B). The strains that showed the best fitting in this matrix were *L. paracasei* L and *L. plantarum* LP82, which reached respectively 9.90 ± 0.03 and 9.74 ± 0.23 log CFU/g at the end of the incubation period. The one that grew less was *L. sakei* M12A with 8.66 ± 0.16 and 9.13 ± 0.19 log CFU/g at 24 and 48 h, respectively. In general, both yeast and LAB strains considered grew on the chickpea flour matrix in line with what reported by (De Pasquale et al., 2020). Since this type of matrix can also harbour degradative and spoilage bacteria, the *Enterobacteriaceae* count was determined (Table S1). The initial *Enterobacteriaceae* load in the matrix was 3.31 ± 0.17 log CFU/g. At the end of fermentation, only samples inoculated with LAB showed counts below 4.00 log CFU/g, with the lowest values observed in the samples containing *L. plantarum* LP23 and *L. curvatus* BS3 (<1 log CFU/g). In contrast, samples inoculated with yeasts had *Enterobacteriaceae* concentrations ranging from 6.50 to 9.25 log CFU/g. Although current EU regulations (EC, 2073/2005) (Commission Regulation, 2005) do not specify microbiological limits for *Enterobacteriaceae* in flours, values above 6–7 log CFU/g, as observed in our yeast-fermented samples, are considered high based on industrial practice and technical guidelines, where ≤3 log CFU/g is typically recommended. Such elevated counts may raise safety concerns due to the potential presence of opportunistic pathogens, including *Escherichia coli*. Further optimization of fermentation parameters, such as acidification kinetics, incubation temperature, and fermentation time, may contribute to a more effective reduction of *Enterobacteriaceae* counts. In fact, one of the main factors contributing to the observed difference is the pH reduction in samples containing LAB, which decreased to 4.3–3.8, compared to those inoculated with yeasts, where the pH stabilized at about 4.9 (Table S2). This is expected, as LAB produce organic acids, such as lactic acid, which lower the pH of the matrix (Wang et al., 2021), while yeasts typically convert sugars into alcohols and carbon dioxide (Maicas, 2023) or perform aerobic metabolism. The sample inoculated with *Y. lipolytica* PO17, a strictly aerobic yeast, showed a slight pH reduction to 5.9 after 48 h, followed by an increase to pH 6.2 after 72 h. This pH increase can be attributed to the yeast's metabolic activity; *Y. lipolytica* can either consume organic acids (such as lactic acid) through respiration or utilize nitrogenous sources, both of which may contribute to the overall pH increase (Gottardi et al., 2021). The presence of pathogenic bacteria, such as *B. cereus*, *L. monocytogenes* and *Salmonella* spp., was also evaluated, but the analyses confirmed their absence.

### 3.2 Protein characterization and peptide content

Since chickpea is an important source of proteins, their modifications in the samples were evaluated using different approaches. The soluble protein content of all the samples expressed in mg BSA eq/g was evaluated using the Bradford assay (Table 2). The control sample exhibited the highest soluble protein content (201.7 mg/g) prior to fermentation, consistent with the characteristics of the original matrix. Following inoculation with microorganisms, a general reduction in soluble protein levels was observed. Among the samples, those inoculated with yeasts presented significantly higher soluble protein values, with *Y. lipolytica* PO17 maintaining the highest concentration (127.8 mg/g), while *S. cerevisiae* FB2 showed the lowest one (45.2 mg/g). In samples fermented by LAB, the lowest concentration was recorded for *L. plantarum* LP23 (3.7 mg/g). The observed changes in soluble protein content before and after fermentation of chickpea flour can be attributed to the proteolytic activity of bacteria and yeasts. These microorganisms produce enzymes capable of hydrolyzing proteins into smaller proteins, peptides, and amino acids (Chandra-Hioe et al., 2016). In particular, LAB rely on protein-rich substrates since they are auxotrophic for several amino acids (Wiley et al., 2017). To assess the impact of fermentation on the protein profile, SDS-PAGE analysis was performed on the samples (Fig. S1). The protein fraction in chickpea flour primarily consists of globulins (53–60 %), glutenins (19–25 %), albumins (8–12 %), and prolamins (3–7 %) (Day, 2013). This composition is reflected in the SDS-PAGE fingerprint, showing bands corresponding to convicilin (∼72 kDa), legumin (∼60 kDa), vicilin (13–19, 30–35, and 50 kDa), and albumin (∼10 kDa). Under reducing conditions, disulfide bonds stabilizing legumin monomers are cleaved, resulting in bands for acidic (∼40 kDa) and basic (∼20 kDa) legumin subunits, consistent with previous reports (Chang et al., 2022). The ∼96–98 kDa band is attributed to lipoxygenase, while proteins at ∼15, 19, 33–37, 50, and 70 kDa are identified as vicilin subunit 7S storage proteins (Chang et al., 2022). Proteins at ∼22–24 and 40 kDa correspond to β and α legumin subunits of 11S proteins (Chang et al., 2022), (Xu et al., 2024). In the control sample, the clearer and more defined bands were those at 20, 37–40 and 50–100 kDa. Fermentation with yeasts reduced the intensity of bands above 75 kDa and around 37–40 kDa. LAB inoculation similarly decreased high-molecular-weight bands, with a notable formation of new bands between 20 and 25 kDa. The most pronounced changes were observed in samples inoculated with *Y. lipolytica* Y3 and *L. plantarum* LP23, which exhibited reduced intensity across all bands. The proteolytic activity of *Y. lipolytica* and *L. plantarum*, even if strain dependent, is well-documented (Nicaud, 2012), (Satılmış et al., 2023). Proteases hydrolyze proteins into smaller molecules, diminishing band intensity in SDS-PAGE. As reported in the study of (Liu et al., 2023), fermentation promotes the breakdown of some indigestible components of the globulin 7S subunits and the release of small peptides and amino acids from macromolecular proteins. This process simplifies enzymatic hydrolysis during human digestion, enhancing the bioavailability of chickpea proteins.

**Table 2:** Soluble proteins, peptides, sugars, phytic acid and total phenol content of not-inoculated (Control T0) and inoculated chickpea flour with *Y. lipolytica* Y3 (Y3), *Y. lipolytica* PO17 (PO17), *D. hansenii* Y15A (Y15A), *D. hansenii* Y17A (Y17A), *S. cerevisiae* FB2 (FB2), *L. plantarum* LP23 (LP23), *L. plantarum* LP82 (LP82), *L. lactis* LBG2 (LBG2), *L. paracasei* L (L), *L. sakei* M12A (M12A) and *L. curvatus* BS3 (BS3) at the end of the incubation period. Results are the average of three replicates (*n* = 3) ± standard deviation. Different letters mean significantly different (*p* < 0.05). - indicates data below the detection limit.

Sample	Soluble proteins (mg BSA eq/g)				Peptides (mg SE/g)				Sugars				Phytic acid (g/100 g)				Total phenol content (g GAE/g)				Sample				Soluble proteins (mg BSA eq/g)			
									D-glucose (mmol/100 g)				Sucrose (mmol/100 g)				RFOs (mmol/100 g)											
Control T0	201.7	±	4.1	^a^	16.2	±	0.5	^e,f^	2.00	±	0.12	^a^	6.42	±	0.32	^c^	1.10	±	0.06	^a^	1.05	±	0.02	^a,b^	1.43	±	0.01	^i^
Y3	78.9	±	0.4	^c^	17.4	±	0.6	^d,e^	1.59	±	0.10	^b^	0.18	±	0.01	^j^	0.18	±	0.01	^h^	0.86	±	0.02	^d^	2.19	±	0.01	^g^
PO17	127.8	±	4.1	^b^	19.6	±	0.1	^a,b,c,d^	1.59	±	0.10	^b^	0.21	±	0.01	^i^	0.03	±	0.00	^j^	0.92	±	0.02	^c,d^	2.58	±	0.01	^d^
Y15A	65.6	±	3.2	^d^	20.3	±	1.5	^a,b,c^	1.62	±	0.10	^b^	0.23	±	0.01	^h^	0.26	±	0.01	^g^	0.82	±	0.02	^d^	2.32	±	0.01	^f^
Y17A	71.1	±	1.4	^d^	17.8	±	0.1	^c,d,e^	1.67	±	0.10	^b^	0.18	±	0.01	^j^	0.54	±	0.03	^c^	0.87	±	0.03	^d^	2.08	±	0.02	^h^
FB2	45.2	±	0.4	^e^	20.7	±	1.2	^a,b^	1.59	±	0.10	^b^	0.18	±	0.01	^j^	–				1.00	±	0.03	^b,c^	2.79	±	0.01	^b,c^
LP23	3.7	±	1.7	^h^	12.2	±	1.5	^g^	1.62	±	0.10	^b^	0.39	±	0.02	^g^	0.46	±	0.02	^e^	1.15	±	0.01	^a^	2.44	±	0.01	^e^
LP82	13.5	±	0.8	^g^	17.6	±	0.5	^d,e^	1.59	±	0.10	^b^	7.14	±	0.36	^a^	0.10	±	0.01	^i^	1.07	±	0.03	^a,b^	2.74	±	0.01	^c^
LBG2	79.7	±	0.2	^c^	21.8	±	0.4	^a^	1.82	±	0.11	^a,b^	2.21	±	0.11	^e^	0.91	±	0.05	^b^	1.08	±	0.01	^a,b^	2.81	±	0.01	^b^
L	4.1	±	2.5	^h^	14.3	±	0.1	^f,g^	1.57	±	0.09	^b^	6.98	±	0.35	^b^	–				1.05	±	0.01	^a,b^	2.37	±	0.03	^f^
M12A	33.1	±	1.1	^f^	19.0	±	0.2	^b,c,d^	1.77	±	0.11	^a,b^	1.57	±	0.08	^f^	0.31	±	0.02	^f^	0.98	±	0.04	^b,c^	2.96	±	0.02	^a^
BS3	17.4	±	1.6	^g^	20.0	±	0.7	^a,b,c,d^	1.59	±	0.10	^b^	3.41	±	0.17	^d^	0.49	±	0.02	^d^	1.04	±	0.04	^b^	2.79	±	0.01	^b,c^

Incubation with microorganisms demonstrated also a significant modification of the peptide content, which was found to be dependent on the strain considered. Results are reported in Table 2. The control sample exhibited a peptide content of 16.2 mg SE/g, which was significantly lower than the one observed in the sample inoculated with *L. lactis* LBG2 (21.8 mg SE/g), while it was significantly higher than the one measured in the sample with *L. plantarum* LP23 (12.2 mg SE/g). Indeed, it is well known that several *L. plantarum* strains have a potent set of enzymes, including cell envelope–bound proteinases and intracellular peptidases, that increase low-molecular-weight peptide bands, detected by electrophoresis, free amino acids and precursor of volatile compounds (Duan et al., 2019). These findings indicate that microbial proteases both produce and consume small peptides during incubation, as noted in previous studies (Emkani et al., 2022). As mentioned earlier, the nutritional needs of LAB can lead to further hydrolysis and consumption of nitrogen sources, reducing peptide content. Nonetheless, an increase in peptides is desirable because some of them can have proven biofunctional benefits, including antioxidant, antihypertensive, hypocholesterolemic, and antimicrobial activities, based on their sequence (Coda et al., 2012), (Real Hernandez and Gonzalez De Mejia, 2019). The results for protein and peptide fractions were consistent across analyses. The control sample showed the highest soluble protein concentration, reflected in both the more prominent SDS-PAGE bands and higher quantified values. Biotechnological processes led to an overall reduction in high-molecular-weight proteins, which were converted into peptides or amino acids. For instance, the sample inoculated with *Y. lipolytica* Y3 had moderate levels of soluble proteins and peptides, as indicated by the relatively faint gel bands. In contrast, *Y. lipolytica* PO17 exhibited a significantly higher soluble protein content compared to other yeast-inoculated samples, confirmed by more intense gel bands. Similarly, the sample fermented by *S. cerevisiae* FB2 showed clear, well-defined bands. Among LAB-fermented samples, the one with *L. plantarum* LP23 resulted in the lowest soluble protein and peptide content. Conversely, *L. lactis* LBG2 produced the highest soluble protein and peptide levels, as evidenced by the strong intensity of its gel bands.

### 3.3 Sugar concentration

A key objective of this study was to reduce the sugar content in chickpea flour, particularly RFOs, which are undesirable due to their role in causing flatulence when metabolized by the gut microbiota of humans and other monogastric animals. Certain microorganisms can lower sugar levels during fermentation by metabolizing complex carbohydrates through specific enzymes (Patrignani et al., 2013). For this reason, glucose, sucrose and RFOs concentrations were estimated before and after fermentation. D-glucose content was 2.00 ± 0.10 mmol/100 g before fermentation, in line with results found by (Gangola et al., 2014). This sugar was significantly reduced by all the microorganisms (Table 2), with sample containing *L. paracasei* L presenting the lower value (1.57 mmol/100 g). On the other hand, sucrose was reduced in almost all samples, especially those inoculated with yeasts. In fact, samples inoculated with *Y. lipolytica* Y3 and PO17, *D. hansenii* Y15A and Y17A, and *S. cerevisiae* FB2 exhibited a significant lower sucrose content (approximately 0.20 mmol/100 g) compared to control (6.42 mmol/100 g), which is comparable to the result obtained by (Gangola et al., 2014). In LAB-fermented samples, sucrose reduction was strain-dependent. It is well known, that sucrose, being a disaccharide, should be hydrolysed into the two hexose moieties before its usage by microbial cells (Gottardi et al., 2021), (Marques et al., 2016). Sucrose naturally present in chickpea flour may be released through the enzymatic hydrolysis of complex sugars by plant-derived enzymes (Marques et al., 2016). However, microorganisms, especially yeasts, possess invertases (β-fructofuranosidases), the enzymes that breakdown sucrose into glucose and fructose (Motta et al., 2023). Consequently, the reduction observed in samples containing yeasts may be attributed to a mutual activity of endogenous enzymes and microorganisms. An interesting result was the reduction in RFOs content in the inoculated samples compared to the control (Table 2). In fact, the initial content measured in the control (1.10 ± 0.06 mmol/100 g), which was in line with the data reported by (Gangola et al., 2014), was below the limit of detection in the samples inoculated with *S. cerevisiae* FB2 and *L. paracasei* L. Previous studies have shown that *S. cerevisiae* significantly reduces oligosaccharides in legume-based matrices (Romero-Espinoza et al., 2020). Oligosaccharides content decreases by 51–98 % compared to the control, as well documented also by (Azeke et al., 2005), which showed a 90 % RFOs reduction during seed fermentation by *L. plantarum*. Similar results were reported by (Galli et al., 2019), where LAB fermentation significantly lowered sugar levels in chickpea flour, primarily due to the α-galactosidase activity. Apart from the two strains mentioned above, yeast-fermented samples generally showed lower values than those obtained with LAB. This trend may be related to enzyme localization: in yeasts, α-galactosidases are often secreted extracellularly (Viana et al., 2006), enabling direct hydrolysis of raffinose-family oligosaccharides, whereas in LAB the enzymes are mainly intracellular or cell-associated (Yoon and Hwang, 2008), limiting their accessibility to the substrate within a complex flour matrix. On the other hand, variability among strains, even within the same species, is widely recognized (Zartl et al., 2018). Nevertheless, it cannot be excluded that part of the activity observed in the flour matrix may also derive from endogenous microorganisms naturally associated with chickpea flour, which could contribute to the overall hydrolysis of RFOs.

### 3.4 Phytate concentration

Phytic acid is one of the most common anti-nutritional factors in chickpea flour. It serves as a primary source of inositol and storage phosphorus in plant seeds. However, phosphorus in this form is inaccessible to monogastric animals due to their lack of endogenous phytases, enzymes that specifically dephosphorylate phytic acid. Additionally, the strong chelating properties of phytic acid reduce the bioavailability of other essential nutrients, such as minerals, proteins, and amino acids (Nissar et al., 2017). For this reason, the potential of the selected strains to reduce phytic acid content in a synthetic medium was evaluated. Results (Fig. S2) showed that both yeasts and LAB reduced the phytic acid content, with the first having a stronger potential (range 16.8–26.7 %) compared to LAB which possessed a species- and strain-specific behaviour (range 5.9 and 20.8 %). Subsequently, phytic acid content was quantified in the food matrix before and after fermentation (Table 2). The control sample had a phytic acid concentration of 1.05 ± 0.02 g/100 g, consistent with the 1.21 g/100 g reported by (Sreerama et al., 2012). Both yeasts and LAB produced strain-specific reductions relative to the control, although the effect was significant only for yeasts. In particular, *Y. lipolytica* and *D. hansenii* strains significantly lowered phytic acid levels, reaching 0.82 g/100 g with *D. hansenii* Y15A. A similar capacity of *Y. lipolytica* to degrade phytic acid has been previously described during okara fermentation (Vong et al., 2018). The lower effectiveness observed in LAB-fermented samples, both *in vitro* and in the food matrix, may be linked to enzyme localization: yeast phytases are predominantly extracellular (e.g., (Georgiev et al., 2024), (Tinrat and Jiraprasertwong, 2025), (Capusoni et al., 2021)), whereas LAB phytases (including those of *L. plantarum*, *L. brevis*, and *L. fermentum*) are mainly intracellular or cell-associated (Sharma et al., 2020) and thus less accessible to the substrate in a complex flour matrix. Furthermore, LAB are typically adapted to nutrient-rich environments with low phytate availability, which has reduced the evolutionary pressure to maintain highly efficient extracellular phytases (Mukhametzyanova et al., 2012).

### 3.5 Antioxidant activity and total phenolic content

The antioxidant activity of the samples was evaluated using both the DPPH and ABTS assays. As reported in Table 3, the antioxidant activity, expressed as mg TE/g of sample, determined by the ABTS assay, revealed that all inoculated samples, especially those with LABs, had a significantly higher antioxidant activity than the control (0.46 mg TE/g). The sample inoculated with *L. paracasei* L exhibited the highest antioxidant activity (1.39 mg TE/g) among LABs, while only the samples inoculated with *S. cerevisiae* FB2 (1.42 mg TE/g) and *Y. lipolytica* PO17 (1.02 mgTE/g) showed significantly higher values than control. These results indicate that antioxidant activity is strain-dependent, with each inoculated sample showing distinct levels of activity. The results of the DPPH assay were partially consistent with those of the ABTS assay. This can be expected due to the different nature of the assay. As reported in Table 3, the DPPH assay indicated that samples inoculated with LABs presented a significantly higher antioxidant activity compared to control (0.06 mg TE/g) and samples containing yeasts. The sample inoculated with *L. paracasei* L demonstrated the highest radical scavenging activity (0.17 mg TE/g), while the sample inoculated with *L. curvatus* BS3 showed the lowest activity (0.12 mg TE/g). Among samples inoculated with yeasts, only the one with *S. cerevisiae* FB2 showed values (0.12 mg TE/g) comparable to LAB, whereas the lowest activity was found in the sample inoculated with *Y. lipolytica* Y3 (0.06 mg TE/g). Fermentation can allow the release or the production of compounds with antioxidant activity. Other than amino acids, and proteins, also phenols present in the matrix may exert an effect. In Table 2, the total phenolic content, expressed as mg GAE/g, is reported. The value measured in the control sample was 1.43 mg GAE/g, which is consistent with what was reported by (Zhao et al., 2021), where various chickpea species were tested. In that study, the *kabuli* variety, known for its light seed coat, exhibited phenolic content ranging from 1.22 to 1.53 mg GAE/g. Upon inoculation with microorganisms, an increase in total phenolic content was observed, with all inoculated samples exceeding 2 mg GAE/g. Generally, LAB-inoculated samples showed a significantly higher increase in total phenolic content compared to those inoculated with yeasts. For those inoculated with LAB, the highest phenolic content was found in *L. sakei* M12A (2.96 mg GAE/g), while the lowest value in *L. paracasei* L (2.37 mg GAE/g). Among samples inoculated with yeast strains, the highest phenolic content was observed in those inoculated with *S. cerevisiae* FB2 and *Y. lipolytica* PO17, with values of 2.79 and 2.58 mg GAE/g, respectively. Notably, a Pearson correlation test confirmed a significant positive correlation between total phenolic content and ABTS antioxidant activity (*r* = 0.64, p < 0.05), suggesting that phenolic compounds play a key role in the antioxidant potential of fermented samples. In contrast, no significant correlation was observed between total phenolic content and DPPH activity indicating that other compounds or mechanisms may be responsible for the radical scavenging activity detected by this assay. The enhanced activity observed in LAB-fermented samples may, in fact, be attributed to specific metabolites produced through their fermentative metabolism. For instance, the ability of *L. plantarum* to enhance antioxidant activity has been reported by (Skrzypczak et al., 2019).

**Table 3:** Radical scavenging activity (mg TE/g sample) and growth of selected probiotic strains in chickpea flour not-inoculated (Control T0) and inoculated with *Y. lipolytica* Y3 (Y3), *Y. lipolytica* PO17 (PO17), *D. hansenii* Y15A (Y15A), *D. hansenii* Y17A (Y17A), *S. cerevisiae* FB2 (FB2), *L. plantarum* LP23 (LP23), *L. plantarum* LP82 (LP82), *L. lactis* LBG2 (LBG2), *L. paracasei* L (L), *L. sakei* M12A (M12A) and *L. curvatus* BS3 (BS3) at the end of the incubation period. Results are the average of three replicates (*n* = 3) ± standard deviation. Different letters mean significantly different (*p* < 0.05).

Sample	Radical scavenging activity								Probiotic strains growth																							
	DPPH (mg TE/g)				ABTS (mg TE/g)				*B. longum* subsp. *infantis* DSM 20088												*L. rhamnosus* GG											
									3 h				6 h				24 h				3 h				6 h				24 h			
Control T0	0.06	±	0.01	^d^	0.46	±	0.06	^c^	6.6	±	0.2	^d,e^	7.8	±	0.5	^a,b^	8.9	±	0.0	^f^	6.52	±	0.3	^c^	7.3	±	0.3	^d^	8.9	±	0.0	^a,b^
Y3	0.06	±	0.01	^d^	0.74	±	0.07	^b,c^	7.6	±	0.2	^a,b,c^	7.9	±	0.4	^a,b^	8.8	±	0.0	^h^	7.64	±	0.3	^a,b,d^	8.2	±	0.2	^a,b,c,d^	8.9	±	0.0	^a,b^
PO17	0.08	±	0.00	^d^	1.02	±	0.20	^a,b^	6.4	±	0.2	^e^	7.7	±	0.3	^a,b^	8.7	±	0.0	^i^	7.09	±	0.1	^b,c,d^	7.9	±	0.8	^a,b,c,d^	8.3	±	0.4	^c^
Y15A	0.08	±	0.00	^d^	0.79	±	0.17	^b,c^	8.4	±	0.3	^a^	8.2	±	0.4	^a,b^	8.8	±	0.0	^g^	7.98	±	0.1	^a,b^	8.8	±	0.0	^a^	8.3	±	0.1	^c^
Y17A	0.07	±	0.01	^d^	0.76	±	0.06	^b,c^	8.4	±	0.5	^a^	8.8	±	0.3	^a^	8.7	±	0.0	^j^	8.14	±	0.3	^a^	8.8	±	0.0	^a,b^	8.4	±	0.4	^b,c^
FB2	0.12	±	0.00	^c^	1.42	±	0.17	^a^	7.8	±	0.1	^a,b^	8.3	±	0.7	^a,b^	8.6	±	0.0	^k^	7.61	±	0.1	^a,b,d^	8.4	±	0.6	^a,b,c^	9.0	±	0.0	^a^
LP23	0.13	±	0.01	^b,c^	1.12	±	0.09	^a,b^	7.1	±	0.0	^b,c,d,e^	7.6	±	0.0	^b^	9.0	±	0.0	^e^	6.64	±	0.5	^c,d^	7.8	±	0.3	^a,b,c,d^	9.1	±	0.0	^a^
LP82	0.15	±	0.00	^a,b^	1.25	±	0.17	^a^	7.6	±	0.1	^a,b,c^	7.6	±	0.8	^b^	9.2	±	0.0	^c^	7.14	±	0.0	^b,c,d^	7.6	±	0.2	^c,d^	9.1	±	0.0	^a^
LBG2	0.13	±	0.00	^b,c^	1.05	±	0.11	^a,b^	6.7	±	0.0	^c,d,e^	7.3	±	0.0	^b^	9.2	±	0.0	^a^	6.61	±	0.0	^c,d^	7.7	±	0.4	^b,c,d^	9.3	±	0.0	^a^
L	0.17	±	0.01	^a^	1.39	±	0.20	^a^	6.7	±	0.3	^d,e^	7.3	±	0.3	^b^	9.2	±	0.0	^b^	7.25	±	0.4	^a,b,c,d^	7.9	±	0.4	^a,b,c,d^	9.1	±	0.0	^a^
M12A	0.14	±	0.00	^b,c^	1.06	±	0.22	^a,b^	7.4	±	0.1	^b,c,d^	7.7	±	0.1	^a,b^	9.1	±	0.0	^d^	7.17	±	0.0	^b,c,d^	8.0	±	0.3	^a,b,c,d^	8.9	±	0.0	^a^
BS3	0.12	±	0.01	^c^	0.72	±	0.07	^b,c^	6.6	±	0.2	^d,e^	7.7	±	0.4	^a,b^	8.6	±	0.0	^k^	6.77	±	0.0	^c,d^	7.6	±	0.1	^c,d^	9.1	±	0.0	^a^

### 3.6 Prebiotic activity

The ability of samples to support probiotics viability and promote their growth was evaluated on two selected probiotic strains (Table 3). After 3 h, the cell load of both *B. longum* subsp. *infantis* DSM 20088 and *L. rhamnosus* GG was significantly higher in samples inoculated with *D. hansenii* compared to the control. After 6 and 24 h of incubation, there were no significant differences among the control and most of the inoculated samples. The use of chickpea flour as a prebiotic has already been demonstrated (Hussein et al., 2020). In addition, it has been shown that the application of some selected microorganisms on chickpea flour can have a higher positive impact on the survival rate of probiotic strains. This is a promising result, although a good source of prebiotics in chickpea flour is usually the presence of RFOs (Hussein et al., 2020), which were reduced in this study thanks to the incubation with the selected yeasts and LAB. This suggests that other compounds, either newly formed or made more available in the matrix, may have contributed synergistically to the observed prebiotic effect. Carbohydrates previously identified in chickpeas, such as sugar alcohols, fructooligosaccharides, resistant starch, and amylose, have demonstrated prebiotic potential (Siva et al., 2020). Yeasts like *D. hansenii* and *Y. lipolytica* are also known to produce sugar alcohols such as xylitol and erythritol/mannitol, respectively (Liang et al., 2023). In particular, xylitol have showed to support probiotic growth (Xiang et al., 2021). In addition, protein hydrolysis during fermentation, evidenced by increased peptide content and SDS-PAGE analysis, may have released peptides with prebiotic activity (Gao et al., 2024). These considerations support the hypothesis that the prebiotic activity observed in fermented chickpea flour is the result of a synergistic contribution of multiple compounds, rather than RFOs alone.

### 3.7 Volatile molecule profiles

**Fig. 2:**
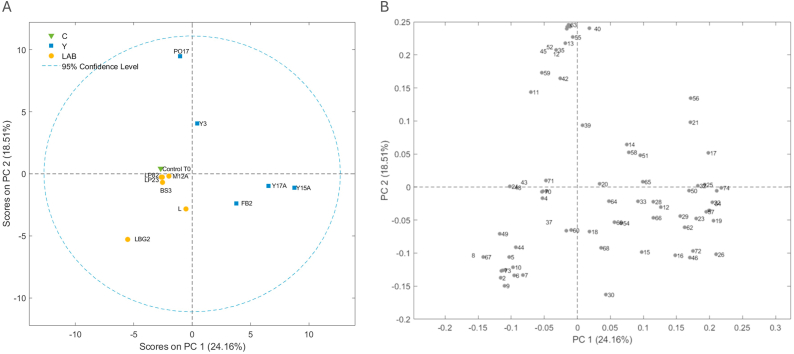
Score plot (A) and loading plot (B) obtained by PCA elaboration of the volatile organic compounds that characterize chickpea flour not-inoculated (Control T0) and inoculated with *Y. lipolytica* Y3 (Y3), *Y. lipolytica* PO17 (PO17), *D. hansenii* Y15A (Y15A), *D. hansenii* Y17A (Y17A), *S. cerevisiae* FB2 (FB2), *L. plantarum* LP23 (LP23), *L. plantarum* LP82 (LP82), *L. lactis* LBG2 (LBG2), *L. paracasei* L (L), *L. sakei* M12A (M12A) and *L. curvatus* BS3 (BS3) at the end of the incubation period. 1: 3-methyl-butanal; 2: Hexanal; 3: Heptanal; 4: 3-methyl-2-butenal; 5: 2-hexenal; 6: 2-heptenal; 7: Nonanal; 8: 2-octenal; 9: Benzaldehyde; 10: Benzeneacetaldehyde; 11: Pentadecanal; 12: Ethanol; 13: 1-propanol; 14: 1-penten-3-ol; 15: 3-methyl-1-butanol; 16: 1-pentanol; 17: 2-heptanol; 18: 2-penten-1-ol; 19: 1-hexanol; 20: 3-hexen-1-ol; 21: 3-octanol; 22: 2-octanol; 23: 1-octen-3-ol; 24: 1-heptanol; 25: Cycloheptanol; 26: 1-octanol; 27: 2,3-butanediol; 28: Cyclooctyl alcohol; 29: 2-octen-1-ol; 30: 1-nonanol; 31: 2-decen-1-ol; 32: Benzyl alcohol; 33: Phenylethyl alcohol; 34: 2,4-decadien-1-ol; 35: 2-butanone; 36: 3-pentanone; 37: 2,3-butanedione; 38: Methyl isobutyl ketone; 39: 2-heptanone; 40: 3-octanone; 41: 2-octanone; 42: Acetoin; 43: 1-methoxy-2-propanone; 44: 2,3-octanedione; 45: 3-ethylcyclopentanone; 46: Acetic acid; 47: Propanoic acid; 48: Butanoic acid; 49: 5-(2-thienyl)pentanoic acid; 50: Hexanoic acid; 51: Nonanoic acid; 52: Linoleic acid ethyl ester; 53: Ethyl Acetate; 54: Butanoic acid, ethyl ester; 55: Pentanoic acid, ethyl ester; 56: Hexanoic acid, ethyl ester; 57: Acetic acid, hexyl ester; 58: Octanoic acid, ethyl ester; 59: 2-Thiopheneacetic acid, tetradecyl ester; 60: 2-Hexenoic acid, ethyl ester; 61: Sulfurous acid, hexyl pentyl ester; 62: 3-methyl-1-butanol acetate; 63: Ethyl oleate; 64: Dodecane; 65: Tetradecane; 66: Hexadecane; 67: D-Limonene; 68: Phenol, 2-methoxy-; 69: Phenol; 70: Acetamide, N-(1-methylpropyl)-; 71: Cyclopentyl acetylene; 72: Furan, 2-pentyl-; 73: 2-Pentenenitrile; 74: Dihydro-5-pentyl-2(3H)-furanone.

With SPME/GC-MS analyses of the not-inoculated (Control T0) and inoculated chickpea flour at the end of the incubation period, around 74 volatile molecules were detected and identified, and are listed in Table S3. The control sample was characterized by alcohols (68.6 %), hydrocarbons (17 %), acids (6.2 %), esters (3.6 %), aldehydes (2.6 %), ketones (1.2 %) and other compounds (0.9 %). These results are slightly different from those obtained by (Zhao et al., 2021), (Khrisanapant et al., 2019) which reported an higher abundance of aldehydes in chickpea flour. The volatile profile of legume-based ingredients depends on many factors such as the cultivar, the hulling technique and the storage method (Karolkowski et al., 2021). Acids and aldehydes can be produced in raw chickpea flour thanks to the activity of both hydroperoxide lyase and lipoxygenase isozymes; then these compounds can be transformed by enzymes like isomerases and alcohol dehydrogenases, resulting in possible unpleasant aromas (Noordraven et al., 2022). The incubation with selected microorganisms determined modifications in relative abundance of some compounds leading to changes in the volatile profile. To understand better the impact of the volatile molecules on sample behaviour a PCA was performed starting from volatile molecules (autoscaled values) (Fig. 2A). A clear separation between yeasts (Y) and LAB samples was observed along PC1 and PC2, explaining respectively 24.16 and 18.51 of the total variance. Most LAB-fermented samples clustered near the control, while yeast-fermented samples, particularly PO17 and Y15A, are clearly separated along PC2 and PC1, respectively, suggesting a distinct volatile profile. The loadings plot (Fig. 2B) allowed to identify the relationships between the PCs and the original variables (volatile compounds) and the correlation between the variables. These results showed that the LAB samples were characterized by a higher overall content of aldehydes and acids, particularly 2-hexenal (5) and hexanal (2) both associated with green and leafy aromas, and organic acids such as hexanoic acid (50, cheesy, fatty), and butanoic acid (48, rancid, sour, buttery), all of which contribute to a more intense and sharp aroma profile (Zhao et al., 2021), (Noordraven et al., 2022).

On the other hand, yeasts were characterized by a higher level of secondary alcohols and esters, which are generally associated with fruity, floral, and sweet aromatic notes. Specifically, only the samples inoculated with PO17 and Y3 contained compounds such as 2,3-butanediol (27 – buttery, sweet), 3-pentanone (36 – fruity, ether-like), 2-heptanol (17 - slightly sweet green-fruity), 3-ethylcyclopentanone (45 – caramel-like, toasted), 2-octanone (41 – waxy, coconut-like), ethyl oleate (63 – fatty, fruity), and octanoic acid ethyl ester (58 – tropical fruit, pineapple-like). In contrast, samples inoculated with Y15A and Y17A were richer in 1-octanol (26 – floral, citrusy), phenylethyl alcohol (33 – rose-like, honeyed), 3-methyl-1-butanol (15 – banana, malty), 2-octanol (22 – waxy, herbal), butanoic acid ethyl ester (54 – sweet, buttery), acetic acid hexyl ester (57 – green, apple-like), 3-methyl-1-butanol acetate (62 – fruity, banana), and hexanoic acid ethyl ester (56 – sweet, fruity, apple-like) (Karolkowski et al., 2021). These compounds could contribute to the complex and pleasant aromatic profile typical of yeast-driven fermentations. These findings suggest that yeast fermentation enhanced the olfactory complexity of chickpea flour through esterification and the production of higher alcohols. This behaviour is consistent with other traditional yeast-driven fermentations, such as wine, bread, and beer, where yeasts are known to contribute predominantly to aroma through the synthesis of fruity volatile compounds (Tofalo et al., 2020).

### 3.8 Characterization of the samples by FTIR

Spectra obtained by FTIR analysis and pretreated by SNV were reported in Fig. S3. For all the samples, the main spectral features are identified at similar wavelengths. Particularly, the range of 3500-3000 cm^−1^ is related to O-H of hydroxyl groups in adsorbed water (stretching vibration), the characteristic peak at 2925 cm^−1^ was ascribed to C-H stretching vibrations, reflecting the presence of lipids and their inclusion complexes with amylose chains. The absorption peaks at about 2960, 2850 and 1750 cm^−1^ reflect asymmetric stretching vibrations of the functional groups -CH3 and -CH2, commonly linked to fatty acids. The bands at about 1640 and 1540, commonly referred to as the Amide I and Amide II, are due to the stretch of the C=O group of the protein peptides and the NH bending and secondarily from the effect of CN stretch, respectively (Garcia-Valle et al., 2021). Finally, the range of 1200-750 cm^−1^ is related to carbohydrates and polysaccharides, while it is known that 994 cm^−1^ is a characteristic absorption peak of glycosidic links of sucrose (Wang et al., 2010).

**Fig. 3:**
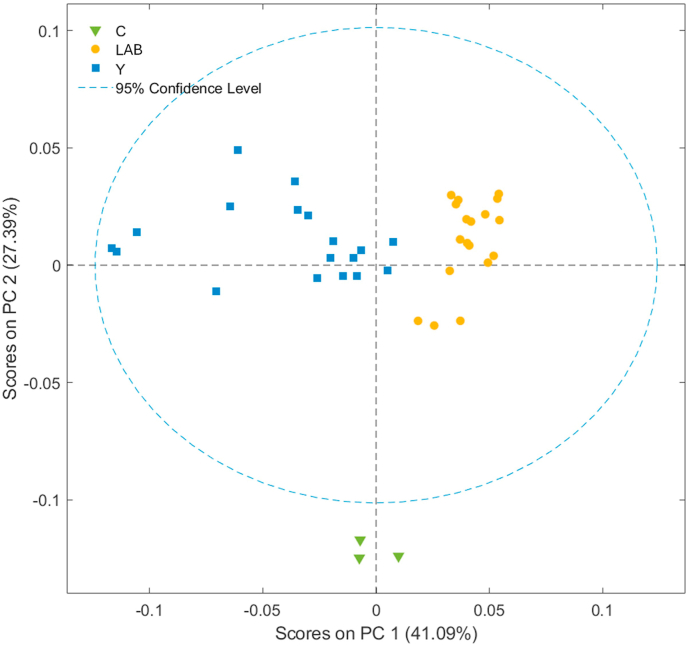
Score plot obtained by the PCA developed considering all the FT-IR spectra.

PCA was used as an explorative technique to visualize the samples based on their similarities in composition. The score plot obtained by the PCA developed considering all the spectral range is shown in Fig. 3. A good samples separation, along the PC1 (41.09 %) and PC2 (27.39 %), was observed according to the strain type and control, suggesting that there are many compositional differences between samples.

**Fig. 4:**
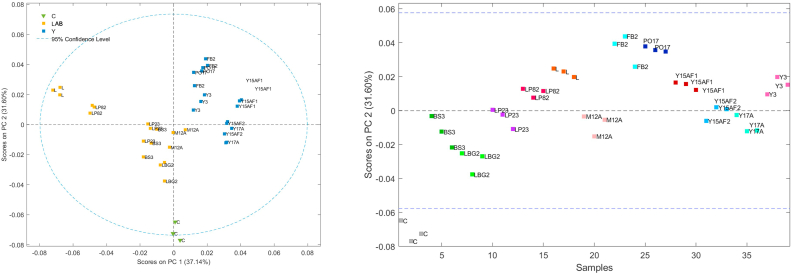
Score plots obtained by the PCA developed considering the FT-IR region between 750 and 1200 cm^−1^.

Subsequently, to evaluate the differences in terms of carbohydrates and polysaccharides, only the region between 1200 and 750 cm^−1^ was subjected to PCA. Score plot (PC1 *vs* PC2) (Fig. 4) combined with the loadings evaluation, suggest that the control sample was characterized by the higher amount of carbohydrates and polysaccharides. Furthermore, evaluating the PC2 scores, it is possible to confirm that LBG2 was the treated sample with the less carbohydrates and polysaccharides reduction, followed by BS3, LP23, M12A, and Y17A. Samples inoculated with FB2, L and PO17 are placed into the opposite side of the control samples, suggesting that are poor of carbohydrates and polysaccharides.

**Fig. 5:**
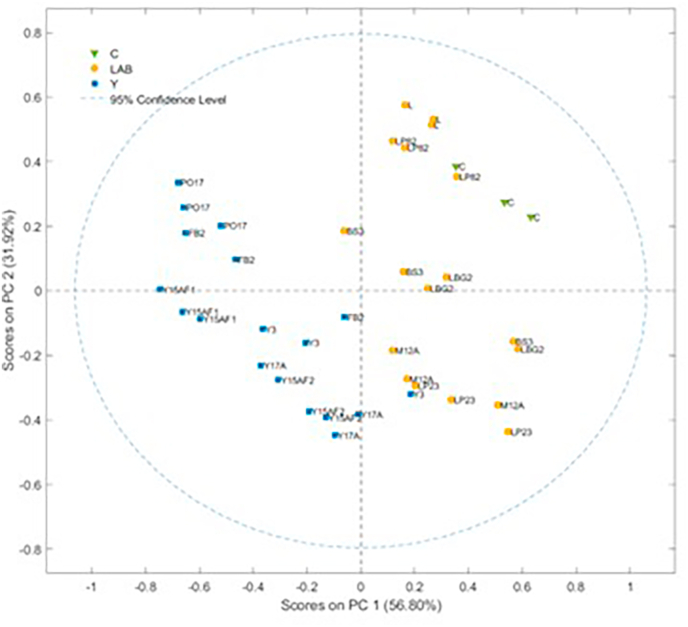
Score plots obtained by the PCA developed considering the FT-IR region between 1060 and 960 cm^−1^.

Evaluating the first derivative of the range between 1060 and 960 cm^−1^ (sucrose), the presence of a pronounced absorption peak at around 995 cm^−1^ was identified only for LP82, L and control, suggesting that these samples were characterized by high amount of sucrose. PCA (score plot PC1 *vs* PC2), according with the data reported in Table 2, confirm that the samples inoculated with yeasts are poor in sucrose respect to those inoculated with 10.13039/100026099LAB (Fig. 5) supporting what observed with the previous analyses.

### 3.9 Preliminary odour evaluation

Odour evaluation is among the main parameters influencing consumer acceptability. In this preliminary evaluation (Fig. S4), we observed that five out eleven samples (*Y. lipolytica* Y3, *D. hansenii* Y15A and Y17A, *L. plantarum* LP82 and *L. curvatus* BS3) were appreciated more than the control. These findings suggest that microbial fermentation, depending on the strain applied, can positively modulate the aromatic characteristics of chickpea flour. The olfactory improvements observed in certain samples may be linked to specific volatile compounds produced during fermentation, including esters and higher alcohols known for their pleasant, fruity notes. However, correlation analysis between the odour descriptors and the volatile compounds detected did not reveal any statistically significant relationships. This may be due to the complexity of odour perception, which often results from synergistic or masking effects among multiple volatiles, rather than from the presence of single dominant compounds. A review of the existing literature did not identify any studies specifically focused on the sensory or odour evaluation of fermented chickpea flour alone, making direct comparisons with our results challenging. Existing studies generally refer to sensory analyses of chickpea-based formulations or final products incorporating fermented flours, typically produced using LAB or sourdough cultures, where overall acceptability was reported to be improved (Nouska et al., 2023), (Zhang et al., 2022a), (Zhang et al., 2022b), (Mefleh et al., 2022). Our findings contribute to filling this gap by providing preliminary evidence that both yeast and LAB strains may enhance the olfactory attributes of chickpea-based ingredients.

## 4 Conclusions

As global demand for alternative protein sources continues to rise, chickpea flour represents a promising ingredient for functional food formulations. This study demonstrated that tailored biotechnological processes based on selected yeast strains can effectively enhance the chemical, biofunctional, and nutritional properties of chickpea flour, effects that have been well documented for LAB. In particular, both yeasts and LAB were able to modify the flour matrix, highlighting their potential to tailor its nutritional characteristics, volatilome profile and odour acceptability. Beyond nutritional improvements, some selected strains also enhanced the prebiotic potential of chickpea flour by stimulating the early growth of probiotic microorganisms, suggesting their suitability for developing sustainable processes aimed at gut health applications. Moreover, the results showed that the selected microorganisms metabolized sugars by hydrolyzing complex carbohydrates and utilizing them as energy sources. This reduction in sugar concentration underscores the potential of these biotechnological processes to decrease the presence of flatulence-inducing compounds in pulse-based products. Additionally, the use of specific strains modulated the volatilome, with yeasts promoting the accumulation of esters and alcohols that contributed to a richer and more pleasant sensory profile. Future research will aim to optimize fermentation conditions (e.g., through response surface methodology on fermentation time and inoculum size) to maximize nutritional and sensory benefits while ensuring product safety. In fact, one limitation of yeast fermentation is the potential for the growth of spoilage or undesired microorganisms. Exploring microbial consortia may offer further advantages, leveraging synergistic interaction, such as acid production by LAB to inhibit spoilage bacteria and ester production by yeasts to enhance aroma. Finally, evaluating the technological performance of this innovative chickpea flour in various food formulations will be essential for its successful industrial application. Overall, these findings pave the way for novel, plant-based products that align with growing consumer demand for sustainable and functional foods.

## CRediT authorship contribution statement

S.A.: Formal analysis, Investigation, Visualization, Writing – original draft, Writing – review & editing; I.G.: Investigation, Writing – review & editing; D.G.: Conceptualization, Formal analysis, Supervision, Validation, Writing – original draft, Writing – review & editing; C.C.: Formal analysis, Software, Validation, Visualization, Writing – review & editing; M.DA.: Writing – review & editing; L.S.: Writing – review & editing; R.L.: Funding acquisition, Project administration, Resources, Writing – review & editing; F.P.: Conceptualization, Project administration, Resources, Validation, Writing – review & editing.

## Funding

This project was funded under the National Recovery and Resilience Plan (NRRP), Mission 4 Component 2 Investment 1.3—Call for tender No. 341 of 15 March 2022 of Italian Ministry of University and Research funded by the European Union—NextGenerationEU; Project code PE00000003, Concession Decree No. 1550 of 11 October 2022 adopted by the Italian Ministry of University and Research, CUP D93C22000890001, Project title "ON Foods—Research and innovation network on food and nutrition Sustainability, Safety and Security—Working ON Foods” and by the Agritech National Research Center and received funding from the European Union Next-GenerationEU (PIANO NAZIONALE DI RIPRESA E RESILIENZA (PNRR)—MISSIONE 4 COMPONENTE 2, INVESTIMENTO 1.4—D.D. 1032 17/06/2022, CN00000022). This manuscript reflects only the authors’ views and opinions; neither the European Union nor the European Commission can be considered responsible for them.

## Declaration of competing interest

The authors declare that they have no known competing financial interests or personal relationships that could have appeared to influence the work reported in this paper.
