# Supplementary material for: Selection of yeasts and lactic acid bacteria to improve the nutritional quality, volatile profile, and biofunctional properties of chickpea flour
Source: Curr Res Food Sci. 2025 Sep 23;11:101204. doi: 10.1016/j.crfs.2025.101204 (PMC12514475; doi:10.1016/j.crfs.2025.101204)
Supplement: Multimedia component 1 [file mmc1.docx]

**Supplementary materials**


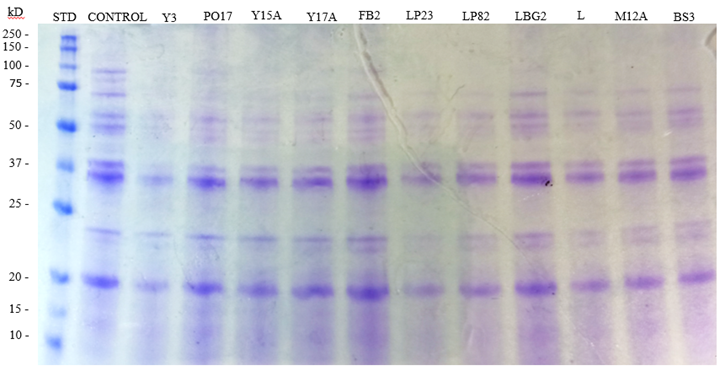


L_s_α

V_s_

C_s_

L_s_β

V_s_f

V_s_f

**Figure S1.** SDS-PAGE electrophoretic profile of chickpea flour, in reducing conditions, not-inoculated (CONTROL) and inoculated with *Y. lipolytica* Y3 (Y3), *Y. lipolytica* PO17 (PO17), *D. hansenii* Y15A (Y15A), *D. hansenii* Y17A (Y17A), *S. cerevisiae* FB2 (FB2), *L. plantarum* LP23 (LP23), *L. plantarum* LP82 (LP82), *L. lactis* LBG2 (LBG2), *L. paracasei* L (L), *L. sakei* M12A (M12A) and *L. curvatus* BS3 (BS3) at the end of the incubation period. C_s_: convicilin subunits; V_s_: vicilin subunits; L_s_α: legumin acidic subunits, L_s_β: legumin basic subunits; V_s_f: vicilin subunit fractions due to post-translational cleavages.


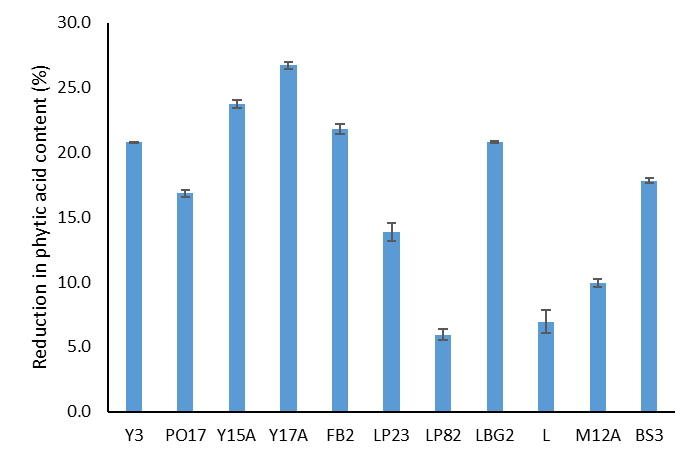


**Figure S2.** *In vitro* reduction of phytic acid by tested strains. Bars show the percentage decrease relative to the initial phytic acid concentration after 48 h incubation in synthetic medium. Data are mean of two technical replicates ± SD (n = 2).

**Figure** **S3.** FT-IR spectra of all the samples (Standard Normal Variate [SNV] pretreatment).

**Figure S4.** Spider plot of olfactory attributes of not-inoculated (Control T0) and inoculated with *Y. lipolytica* Y3 (Y3), *Y. lipolytica* PO17 (PO17), *D. hansenii* Y15A (Y15A), *D. hansenii* Y17A (Y17A), *S. cerevisiae* FB2 (FB2), *L. plantarum* LP23 (LP23), *L. plantarum* LP82 (LP82), *L. lactis* LBG2 (LBG2), *L. paracasei* L (L), *L. sakei* M12A (M12A) and *L. curvatus* BS3 (BS3) chickpea flour at the end of the incubation period. Results are the mean of three independent replicates (*n* = 3).

**Table S1.** Cell loads (log CFU/g) of *Enterobacteriaceae* in the samples of chickpea flour inoculated with yeasts (*Y. lipolytica* Y3 (Y3), *Y. lipolytica* PO17 (PO17), *D. hansenii* Y15A (Y15A), *D. hansenii* Y17A (Y17A) and *S. cerevisiae* FB2 (FB2)) or LAB (*L. plantarum* LP23 (LP23), *L. plantarum* LP82 (LP82), *L. lactis* LBG2 (LBG2), *L. paracasei* L (L), *L. sakei* M12A (M12A) and *L. curvatus* BS3 (BS3)). Cell loads were determined immediately before incubation (0 h) and after 24, 48 h incubation for LAB or 24, 48 and 72 h for yeasts. The results are the mean of three independent replicates (*n* = 3) ± standard deviation.

| ***Enterobacteriaceae* (Log CFU/g)** | | | | | | | | | | | | |
| --- | --- | --- | --- | --- | --- | --- | --- | --- | --- | --- | --- | --- |
|  | **0 h** | | | **24 h** | | | **48 h** | | | **72 h** | | |
| **Y3** | 3.31 | ± | 0.17 | 9.37 | ± | 0.09 | 8.00 | ± | 0.00 | 8.47 | ± | 0.02 |
| **PO17** | 3.31 | ± | 0.17 | 9.32 | ± | 0.03 | 8.69 | ± | 0.09 | 9.25 | ± | 0.09 |
| **Y15A** | 3.31 | ± | 0.17 | 8.22 | ± | 0.14 | 8.17 | ± | 0.18 | 6.48 | ± | 0.35 |
| **Y17A** | 3.31 | ± | 0.17 | 9.42 | ± | 0.12 | 7.52 | ± | 0.11 | 7.80 | ± | 0.47 |
| **FB2** | 3.31 | ± | 0.17 | 7.65 | ± | 0.34 | 5.70 | ± | 0.13 | - | | |
| **LP23** | 3.31 | ± | 0.17 | nd | | |  | nd |  | - | | |
| **LP82** | 3.31 | ± | 0.17 | 7.57 | ± | 0.13 | 1.75 | ± | 0.18 | - | | |
| **LBG2** | 3.31 | ± | 0.17 | 4.92 | ± | 0.15 | 3.67 | ± | 0.21 | - | | |
| **L** | 3.31 | ± | 0.17 | 7.32 | ± | 0.31 | 1.88 | ± | 0.57 | - | | |
| **M12A** | 3.31 | ± | 0.17 | 8.26 | ± | 0.21 | 2.48 | ± | 0.15 | - | | |
| **BS3** | 3.31 | ± | 0.17 | nd | | |  | nd |  | - | | |

-: Not measured

nd: below limit of detection (< 1 log CFU/g)

**Table S2.** pH values of the samples of chickpea inoculated with (*Y. lipolytica* Y3 (Y3), *Y. lipolytica* PO17 (PO17), *D. hansenii* Y15A (Y15A), *D. hansenii* Y17A (Y17A) and *S. cerevisiae* FB2 (FB2)) or LAB (*L. plantarum* LP23 (LP23), *L. plantarum* LP82 (LP82), *L. lactis* LBG2 (LBG2), *L. paracasei* L (L), *L. sakei* M12A (M12A) and *L. curvatus* BS3 (BS3)). Values were registered immediately before incubation (0 h) and after 24, 48 h incubation for LAB or 24, 48 and 72 h for yeasts. The results are the mean of three independent replicates (*n* = 3) ± standard deviation.

|  | **0 h** | | | **24 h** | | | **48 h** | | | **72 h** | | |
| --- | --- | --- | --- | --- | --- | --- | --- | --- | --- | --- | --- | --- |
| **Y3** | 6.5 | ± | 0.1 | 6.1 | ± | 0.0 | 5.8 | ± | 0.0 | 5.0 | ± | 0.0 |
| **PO17** | 6.5 | ± | 0.1 | 6.1 | ± | 0.0 | 5.9 | ± | 0.0 | 6.2 | ± | 0.0 |
| **Y15A** | 6.5 | ± | 0.1 | 5.9 | ± | 0.0 | 4.9 | ± | 0.0 | 4.8 | ± | 0.0 |
| **Y17A** | 6.5 | ± | 0.1 | 5.9 | ± | 0.0 | 5.0 | ± | 0.0 | 4.8 | ± | 0.0 |
| **FB2** | 6.5 | ± | 0.1 | 5.8 | ± | 0.0 | 4.8 | ± | 0.0 | - | | |
| **LP23** | 6.5 | ± | 0.1 | 4.0 | ± | 0.0 | 3.8 | ± | 0.0 | - | | |
| **LP82** | 6.5 | ± | 0.1 | 4.8 | ± | 0.0 | 4.0 | ± | 0.0 | - | | |
| **LBG2** | 6.5 | ± | 0.1 | 4.9 | ± | 0.0 | 4.1 | ± | 0.0 | - | | |
| **L** | 6.5 | ± | 0.1 | 4.4 | ± | 0.0 | 3.9 | ± | 0.0 | - | | |
| **M12A** | 6.5 | ± | 0.1 | 4.8 | ± | 0.0 | 4.3 | ± | 0.0 | - | | |
| **BS3** | 6.5 | ± | 0.1 | 4.3 | ± | 0.0 | 4.1 | ± | 0.0 | - | | |

-: Not measured

**Table S3.** Volatile organic components detected by SPME/GC-MS in chickpea flour not-inoculated (Control T0) and inoculated with *Y. lipolytica* Y3 (Y3), *Y. lipolytica* PO17 (PO17), *D. hansenii* Y15A (Y15A), *D. hansenii* Y17A (Y17A), *S. cerevisiae* FB2 (FB2), *L. plantarum* LP23 (LP23), *L. plantarum* LP82 (LP82), *L. lactis* LBG2 (LBG2), *L. paracasei* L (L), *L. sakei* M12A (M12A) and *L. curvatus* BS3 (BS3) at the end of the incubation period, and expressed as mg/L equivalent. Results are the average of two replicates (*n* = 2). - indicates data below the detection limit.

| **Volatile organic compound** | **Reference number in PCA graph** | **Control T0** | **Y3** | **PO17** | **Y15A** | **Y17A** | **FB2** | **LP23** | **LP82** | **LBG2** | **L** | **M12A** | **BS3** |
| --- | --- | --- | --- | --- | --- | --- | --- | --- | --- | --- | --- | --- | --- |
| 3-methyl-butanal | 1 | - | - | - | - | - | - | - | - | 1.41 | - | - | - |
| Hexanal | 2 | 0.11 | 0.31 | 0.26 | - | - | 0.83 | 0.34 | 0.93 | 6.05 | 1.08 | 1.14 | 0.33 |
| Heptanal | 3 | - | - | - | - | - | - | - | 0.09 | 0.65 | - | 0.12 | 0.05 |
| 3-methyl-2-butenal | 4 | - | - | - | - | - | - | - | - | - | - | 0.19 | 0.03 |
| 2-hexenal | 5 | - | 0.04 | - | - | - | - | - | - | 0.22 | - | 0.03 | - |
| 2-heptenal | 6 | - | 0.11 | 0.16 | - | - | 0.47 | 0.06 | 0.19 | 1.62 | 0.47 | 0.16 | 0.03 |
| Nonanal | 7 | - | - | - | - | - | - | 0.03 | 0.03 | 0.24 | 0.38 | 0.09 | 0.02 |
| 2-octenal | 8 | - | - | - | - | - | - | 0.03 | 0.15 | 1.35 | - | 0.13 | 0.02 |
| Benzaldehyde | 9 | - | - | - | - | - | - | 0.05 | 0.12 | 0.55 | 0.53 | 0.09 | 0.17 |
| Benzeneacetaldehyde | 10 | - | - | - | - | - | - | - | - | 0.62 | - | - | - |
| Pentadecanal | 11 | - | 0.08 | 0.24 | - | - | - | - | - | 0.16 | - | - | - |
| **Total adehydes** |  | 0.11 | 0.55 | 0.67 | - | - | 1.29 | 0.51 | 1.51 | 12.86 | 2.46 | 1.95 | 0.65 |
| Ethanol | 12 | 0.27 | 10.96 | 15.69 | 28.49 | 16.05 | 94.72 | 0.50 | 1.42 | 4.61 | 5.41 | 2.72 | 1.14 |
| 1-propanol | 13 | - | - | 2.50 | - | - | - | - | - | - | - | - | - |
| 1-penten-3-ol | 14 | 0.03 | 0.29 | 0.27 | 0.30 | 0.20 | - | 0.02 | 0.09 | 0.20 | 0.33 | 0.08 | 0.10 |
| 3-methyl-1-butanol | 15 | - | 0.11 | 0.52 | 2.10 | 0.99 | 6.22 | 0.15 | 0.17 | 2.17 | 0.67 | 0.27 | 0.04 |
| 1-pentanol | 16 | 0.14 | 0.35 | 0.38 | 2.20 | 1.80 | 0.50 | 0.18 | 0.41 | 1.03 | 2.10 | 0.35 | 0.29 |
| 2-heptanol | 17 | - | 0.11 | 0.10 | 0.27 | 0.26 | - | - | - | - | - | - | - |
| 2-penten-1-ol | 18 | - | 0.16 | 0.17 | 0.29 | 0.19 | - | 0.08 | 0.15 | 0.29 | 0.64 | 0.13 | 0.13 |
| 1-hexanol | 19 | 2.33 | 3.53 | 6.07 | 39.62 | 27.63 | 8.30 | 1.47 | 4.61 | 6.46 | 12.25 | 2.97 | 2.59 |
| 3-hexen-1-ol | 20 | 0.08 | 0.07 | 0.12 | 0.19 | - | - | 0.03 | - | 0.08 | 0.23 | 0.05 | 0.03 |
| 3-octanol | 21 | - | 0.10 | 0.15 | 0.17 | 0.28 | - | - | - | - | - | - | - |
| 2-octanol | 22 | - | 0.03 | - | 0.18 | 0.14 | - | - | - | - | - | - | - |
| 1-octen-3-ol | 23 | 0.02 | 0.45 | 0.43 | 1.19 | 1.01 | 0.53 | 0.03 | 0.15 | 0.72 | 0.41 | 0.11 | 0.06 |
| 1-heptanol | 24 | 0.05 | - | 0.32 | - | - | - | - | - | 0.57 | - | - | - |
| Cycloheptanol | 25 | - | 0.07 | 0.11 | 0.48 | 0.68 | - | - | - | - | - | - | - |
| 1-octanol | 26 | 0.06 | 0.17 | - | 1.08 | 1.18 | 0.81 | 0.04 | 0.16 | 0.29 | 0.46 | 0.08 | 0.05 |
| 2,3-butanediol | 27 | - | 0.76 | 2.93 | - | - | - | - | - | - | - | - | - |
| Cyclooctyl alcohol | 28 | - | - | - | - | 1.19 | - | - | - | - | - | - | - |
| 2-octen-1-ol | 29 | - | - | - | 1.10 | - | 0.15 | - | - | 0.10 | - | - | - |
| 1-nonanol | 30 | 0.06 | - | - | - | 0.47 | 0.42 | 0.05 | 0.13 | 0.35 | 0.43 | 0.11 | 0.05 |
| 2-decen-1-ol | 31 | - | - | - | 0.29 | 0.30 | - | - | - | - | - | - | - |
| Benzyl alcohol | 32 | - | 0.05 | 0.06 | 0.30 | 0.15 | - | 0.02 | - | 0.06 | - | - | - |
| Phenylethyl alcohol | 33 | 0.02 | 0.15 | 0.82 | 0.50 | 0.51 | 4.01 | 0.02 | - | 0.27 | - | 0.03 | - |
| 2,4-decadien-1-ol | 34 | - | - | - | 0.44 | 0.42 | - | - | - | - | - | - | - |
| **Total alcohols** |  | 3.06 | 17.37 | 30.64 | 79.19 | 53.46 | 115.66 | 2.59 | 7.28 | 17.19 | 22.93 | 6.89 | 4.49 |
| 2-butanone | 35 | - | - | 2.44 | - | - | - | - | - | - | - | 0.80 | - |
| 3-pentanone | 36 | - | 0.37 | 0.64 | - | - | - | - | - | - | - | - | - |
| 2,3-butanedione | 37 | - | - | - | - | - | - | 0.31 | - | - | 1.89 | - | - |
| Methyl isobutyl ketone | 38 | 0.06 | - | - | - | - | - | - | - | - | - | - | - |
| 2-heptanone | 39 | - | 0.12 | - | - | - | - | - | - | - | - | - | - |
| 3-octanone | 40 | - | 0.21 | 0.52 | 0.11 | - | - | - | - | - | - | - | - |
| 2-octanone | 41 | - | 0.04 | 0.13 | - | - | - | - | - | - | - | - | - |
| Acetoin | 42 | - | 0.50 | 4.14 | - | - | - | - | 0.25 | 0.07 | 2.68 | 0.17 | - |
| 1-methoxy-2-propanone | 43 | - | - | - | - | - | - | 0.57 | - | - | - | - | 0.10 |
| 2,3-octanedione | 44 | - | 0.04 | - | - | - | - | - | - | 0.12 | - | - | - |
| 3-ethylcyclopentanone | 45 | - | 0.06 | 0.13 | - | - | - | - | - | - | - | - | - |
| **Total ketones** |  | 0.06 | 1.34 | 7.99 | 0.11 | - | - | 0.88 | 0.25 | 0.19 | 4.58 | 0.97 | 0.10 |
| Acetic acid | 46 | 0.14 | 4.54 | 1.11 | 29.64 | 19.89 | 23.75 | 5.24 | 8.49 | 1.93 | 33.20 | 6.79 | 1.67 |
| Propanoic acid | 47 | - | - | - | - | - | - | - | - | - | - | - | 0.03 |
| Butanoic acid | 48 | - | - | - | - | - | 85.92 | - | 0.59 | - | - | 0.25 | - |
| 5-(2-thienyl)pentanoic acid | 49 | 0.13 | 0.10 | 0.20 | - | - | - | - | 0.20 | 0.55 | 0.51 | - | 0.10 |
| Hexanoic acid | 50 | - | 0.85 | 0.12 | 0.56 | 0.92 | 0.48 | 0.05 | 0.19 | - | 0.74 | 0.09 | 0.05 |
| Nonanoic acid | 51 | - | 0.08 | - | - | 0.09 | - | - | - | - | - | - | - |
| **Total acids** |  | 0.27 | 5.57 | 1.42 | 30.19 | 20.90 | 110.16 | 5.29 | 9.49 | 2.47 | 34.45 | 7.12 | 1.84 |
| Linoleic acid ethyl ester | 52 | - | 0.13 | 0.19 | - | - | - | - | - | - | - | - | - |
| Ethyl Acetate | 53 | - | 2.79 | 1.85 | 10.61 | 3.65 | 6.80 | 0.10 | 0.22 | - | - | 0.21 | - |
| Butanoic acid, ethyl ester | 54 | - | - | - | - | - | 13.60 | - | - | - | - | - | - |
| Pentanoic acid, ethyl ester | 55 | - | 0.09 | 0.08 | - | - | - | - | - | - | - | - | - |
| Hexanoic acid, ethyl ester | 56 | - | 0.53 | 0.45 | 0.57 | 0.31 | 0.31 | - | - | - | - | - | - |
| Acetic acid, hexyl ester | 57 | - | - | - | 3.82 | 1.31 | 0.33 | - | - | - | - | 0.04 | - |
| Octanoic acid, ethyl ester | 58 | - | 0.20 | 0.42 | 0.13 | - | 0.98 | - | - | - | - | - | - |
| 2-Thiopheneacetic acid, tetradecyl ester | 59 | 0.10 | - | 0.24 | - | - | - | - | 0.20 | - | - | - | - |
| 2-Hexenoic acid, ethyl ester | 60 | - | - | - | - | - | - | - | - | - | 0.93 | - | - |
| Sulfurous acid, hexyl pentyl ester | 61 | 0.05 | - | - | - | - | - | - | - | - | - | - | - |
| 3-methyl-1-butanol acetate | 62 | - | - | - | 0.30 | - | 0.27 | - | - | - | - | 0.05 | - |
| Ethyl oleate | 63 | - | 0.20 | 0.39 | - | - | - | - | - | - | - | - | - |
| **Total esters** |  | 0.16 | 3.94 | 3.63 | 15.44 | 5.26 | 22.30 | 0.10 | 0.42 | 0.00 | 0.93 | 0.30 | 0.00 |
| Dodecane | 64 | 0.20 | 0.26 | - | - | - | 0.78 | - | 0.19 | - | - | - | - |
| Tetradecane | 65 | 0.41 | 0.67 | 0.58 | - | 1.17 | 2.61 | - | 0.59 | - | - | - | - |
| Hexadecane | 66 | 0.13 | 0.16 | - | - | 0.55 | 0.86 | - | 0.19 | - | - | - | - |
| D-Limonene | 67 | 0.02 | - | - | - | - | - | 0.08 | - | 0.11 | - | 0.09 | 0.03 |
| **Total hydrocarbons** |  | 0.76 | 1.09 | 0.58 | - | 1.72 | 4.26 | 0.08 | 0.97 | 0.11 | - | 0.09 | 0.03 |
| Phenol, 2-methoxy- | 68 | - | - | - | - | - | 0.43 | - | - | - | 0.38 | 0.12 | - |
| Phenol | 69 | - | 1.88 | 1.90 | 1.16 | 0.58 | 10.46 | 0.04 | 1.43 | - | 9.12 | 2.08 | - |
| **Total phenols** |  | - | 1.88 | 1.90 | 1.16 | 0.58 | 10.88 | 0.04 | 1.43 | - | 9.50 | 2.20 | - |
| Acetamide, N-(1-methylpropyl)- | 70 | - | - | - | - | - | - | - | 0.19 | - | - | - | 0.08 |
| Cyclopentyl acetylene | 71 | 0.04 | - | - | - | - | - | - | - | - | - | - | - |
| Furan, 2-pentyl- | 72 | - | 0.12 | 0.11 | 0.49 | 0.25 | 0.31 | 0.03 | 0.12 | 0.24 | 0.26 | 0.07 | 0.04 |
| 2-Pentenenitrile | 73 | - | - | - | - | - | - | 0.02 | - | 0.23 | - | 0.02 | 0.03 |
| Dihydro-5-pentyl-2(3H)-furanone | 74 | - | 0.11 | 0.11 | 0.67 | 0.38 | 0.16 | - | - | - | - | - | - |
| **Total others** |  | 0.04 | 0.23 | 0.22 | 1.16 | 0.62 | 0.47 | 0.04 | 0.31 | 0.48 | 0.26 | 0.09 | 0.15 |
